# Insights into the occurrence of rabies viruses in multi-species animals based on diagnostic laboratory submissions

**DOI:** 10.1128/spectrum.00419-25

**Published:** 2025-06-12

**Authors:** Aurelle Yondo, Ben Enyetornye, Binu T. Velayudhan

**Affiliations:** 1 Athens Veterinary Diagnostic Laboratory, College of Veterinary Medicine, University of Georgia 70734, Athens, Georgia, USA; Pennsylvania State College of Medicine, Hershey, Pennsylvania, USA

**Keywords:** rabies, multi-species, diagnostic laboratory, retrospective study, animal health

## Abstract

Rabies is a fatal zoonotic disease caused by the rabies virus (RABV), primarily affecting the central nervous system of mammals. Understanding the epidemiology of animal rabies is critical for developing effective prevention and control strategies. This study aimed to analyze animal rabies cases received at a veterinary diagnostic laboratory in Georgia, USA, over 5 years (2019–2023), focusing on the most commonly infected species, seasonality trends, and geographical distributions. A total of 1,560 rabies-suspect cases, representing 21 species of animals, were tested using a direct fluorescent antibody test (DFAT). Of 1,560 cases, 5.6% (88/1560) were positive across 11 species, with domestic animals accounting for 17% (15/88) of rabies cases, whereas wildlife species exhibited a higher occurrence of 83% (73/88). Among wildlife, the affected species were raccoons (35.2% [31/88]), skunks (25% [22/88]), white-tailed deer (8% [7/88]), foxes (6.8% [6/88]), bats (4.5% [4/88]), bobcats (2.3% [2/88]), and great kudu (1.1% [1/88]). In domestic animals, the affected species included bovine (6.8% [6/88]), feline (5.7% [5/88]), caprine (2.3% [2/88]), and equine (2.3% [2/88]). Positive cases were predominantly detected in submissions from Georgia, with a few additional cases identified in neighboring states and unknown locations. Furthermore, fall, spring, and summer seasons showed high infection rates compared with winter. Our findings highlight distinct seasonal trends and the significant burden of rabies among wildlife in the Southeastern United States.

**IMPORTANCE:**

Rabies is a fatal zoonotic viral disease that affects the central nervous system of mammals including humans. It is transmitted mainly through bites or scratches by infected animals such as dogs, bats, raccoons, and other wild animals. The present study analyzed data on clinical specimens submitted to a veterinary diagnostic laboratory for the detection of rabies in domestic and wild animals for a period of 5 years. The study examined a total of 1,560 rabies-suspect cases, representing 21 species of animals tested using the standard direct fluorescent antibody (DFA) assay. Out of 1,560 cases, 5.6% were positive across 11 species, with domestic animals accounting for 17% and wild animals accounting for 83% of the total cases. Different species of wild animal species showed a significantly higher incidence of rabies, highlighting the importance of wildlife in spreading rabies to domestic animals and the threat it poses to public health.

## INTRODUCTION

Rabies is a life-threatening, progressive neurologic viral disease transmitted via the saliva of infected animals, usually through bites or scratches (1–3). It is caused by a bullet-shaped, single-stranded, non-segmented, negative-sense RNA virus belonging to the genus *Lyssavirus* and the *Rhabdoviridae* family (4). The rabies virus (RABV) primarily targets the central nervous system of humans and animals, leading to encephalitis with fatal symptoms, including hyperexcitability, autonomic dysfunction, hydrophobia, and aerophobia after an average incubation period of 20–90 days (3, 5, 6). There are rare cases with longer incubation periods, extending up to years, depending on factors such as exposure site, viral load, and host immune response (7, 8). It is a multiple-host pathogen that affects all warm-blooded animals, but dogs and wildlife serve as significant reservoirs for the virus (9, 10). Rabies represents a significant public health threat on every continent except Antarctica (11), with an estimated 60,000 human cases reported annually (12, 13). Although the global burden of rabies seems to have declined over the past three decades, the disease remains a persistent problem for many countries including developed nations (14). In wildlife, the rabies virus continues to circulate, frequently exposing unvaccinated domestic animals, especially dogs, making control incredibly challenging (15), underscoring the 2030 dog-mediated rabies elimination goals (16).

In the USA, approximately 4,000 animal rabies cases are reported annually, with over 90% occurring in wildlife such as skunks, bats, raccoons, and foxes (17). In 2020, 4,090 wildlife and 389 domestic animals tested positive for rabies in the country (18). Human rabies cases in the Americas and Caribbean have been linked to sporadic spillover from wildlife, as widespread preventive measures, such as vaccination, have been implemented for companion animals (19, 20). Each year, more than 4 million Americans report animal bites with approximately 800,000 seeking medical attention (17). Humans exposed to rabies-positive animals often face long quarantine periods and post-exposure prophylaxis (PEP), causing discomfort and financial burdens to many families (21). Moreover, PEP is expensive and associated with adverse reactions (22). The estimated annual direct and indirect costs of PEP are $1.7 billion and $1.3 billion, respectively (23). This suggests that improving rabies control in wildlife through oral vaccination programs, combined with routine vaccination of companion animals and livestock (24, 25) at a lower cost, could alleviate the burden on animal owners.

Given these challenges, constantly updating the epidemiological trends of animal rabies cases submitted to veterinary diagnostic laboratories is crucial to guide the structuring and implementation of preventive and control measures in animals and provide insights into human exposures to the disease. However, there is limited information on rabies surveillance data in the southeastern United States. This study analyzed animal rabies cases submitted to the Athens Veterinary Diagnostic Laboratory (AVDL, University of Georgia, Athens, GA, USA) from 2019 to 2023. We focused on identifying any patterns in rabies cases, such as the occurrence among wild and domestic species, analyzing seasonal trends, and mapping the geographical distribution of positive cases. Our findings provide useful insights for long-term policy decisions and improving rabies prevention and control strategies.

## MATERIALS AND METHODS

### Data collection and analysis

We queried the Athens Veterinary Diagnostic Laboratory (AVDL) database using the Laboratory Information Management System called VetView to retrieve 1,560 rabies-suspect cases submitted to the laboratory from 2019 to 2023. Tissue samples were submitted by various clients from within and outside Georgia, including the Southeastern Cooperative Wildlife Disease Study (SCWDS). Those specimens were tested using the DFAT, and the data were collected in an Excel spreadsheet. Each case was individually reviewed to capture details including accession number, received date, specimen type, species, geographic locations, seasonality, and diagnostic outcomes. Cases were categorized as positive and negative based on DFAT results. We categorized the positive cases by species, and seasonal trends were analyzed by grouping the data into four seasons (winter, spring, summer, and fall). We examined temporal trends over the 5-year period to identify significant patterns in rabies occurrence. Positive cases were mapped for geographic distribution and further classified into cases originating from wildlife and domestic animals for comparative analysis.

### Direct fluorescent antibody test

According to the World Health Organization (WHO), the DFAT is considered the gold standard for rabies testing, designed to detect the presence of rabies virus (RABV) antigens in brain tissue (26). The rabies testing procedure was performed following the Centers for Disease Control and Prevention guidelines and standards (27). Brain tissue samples were collected and sectioned to include identifiable areas of the right and left lateral lobes of the cerebellum, the vermis, and the brainstem. Tissue impressions were prepared on clean glass microscope slides. These slides were then air-dried and fixed in cold acetone (−20°C) for at least an hour to preserve antigen integrity. After the fixation period, the slides were stained with three separate conjugates, including the EMD Millipore Corporation 5100 Light Diagnostics Rabies DFA Reagent (EMD Millipore Corp, Temecula, CA), Fujirebio Diagnostics Inc FITC Anti-Rabies Monoclonal Globulin (FDI, Malvern, PA), and the Millipore Light Diagnostics Rabies Negative Control, Monoclonal Antibody FITC Conjugate 5102 (EMD Millipore Corp, Temecula, CA) conjugates. Stained slides were incubated in the humid chamber for 30 min, allowing sufficient time for antibody-antigen binding. After incubation, slides were rinsed with rabies phosphate-buffered saline (PBS) to remove unbound antibodies and examined under a fluorescent microscope’s FITC filter. The interpretation of slides was based on fluorescence intensity and antigen distribution. Positive rabies impression smears showed a bright apple-green fluorescence in rabies virus-infected neuronal cells represented by massive intracytoplasmic inclusions of various shapes (dust-like particles, large, round to oval). In all observed fields, samples considered negative displayed no fluorescence and no inclusions, and the tissue appeared as a dull red background. Before any testing, conjugates were subjected to an initial titration to determine the optimal working dilution for routine use. We prepared serial dilutions of conjugates that will be tested with control material from naturally infected animals. Brain tissues used were from previously submitted accessions, particularly a raccoon strain that was tested and confirmed rabies-positive to ensure the reliability of the results.

### Statistical analysis

The statistical analysis was conducted using JMP Pro version software (https://www.jmp.com/en/software/data-analysis-software). χ^2^ tests were used to evaluate significant associations between the species, seasonality, and rabies occurrence with significance determined at a *P*-value < 0.05.

## RESULTS

**Fig 1:**
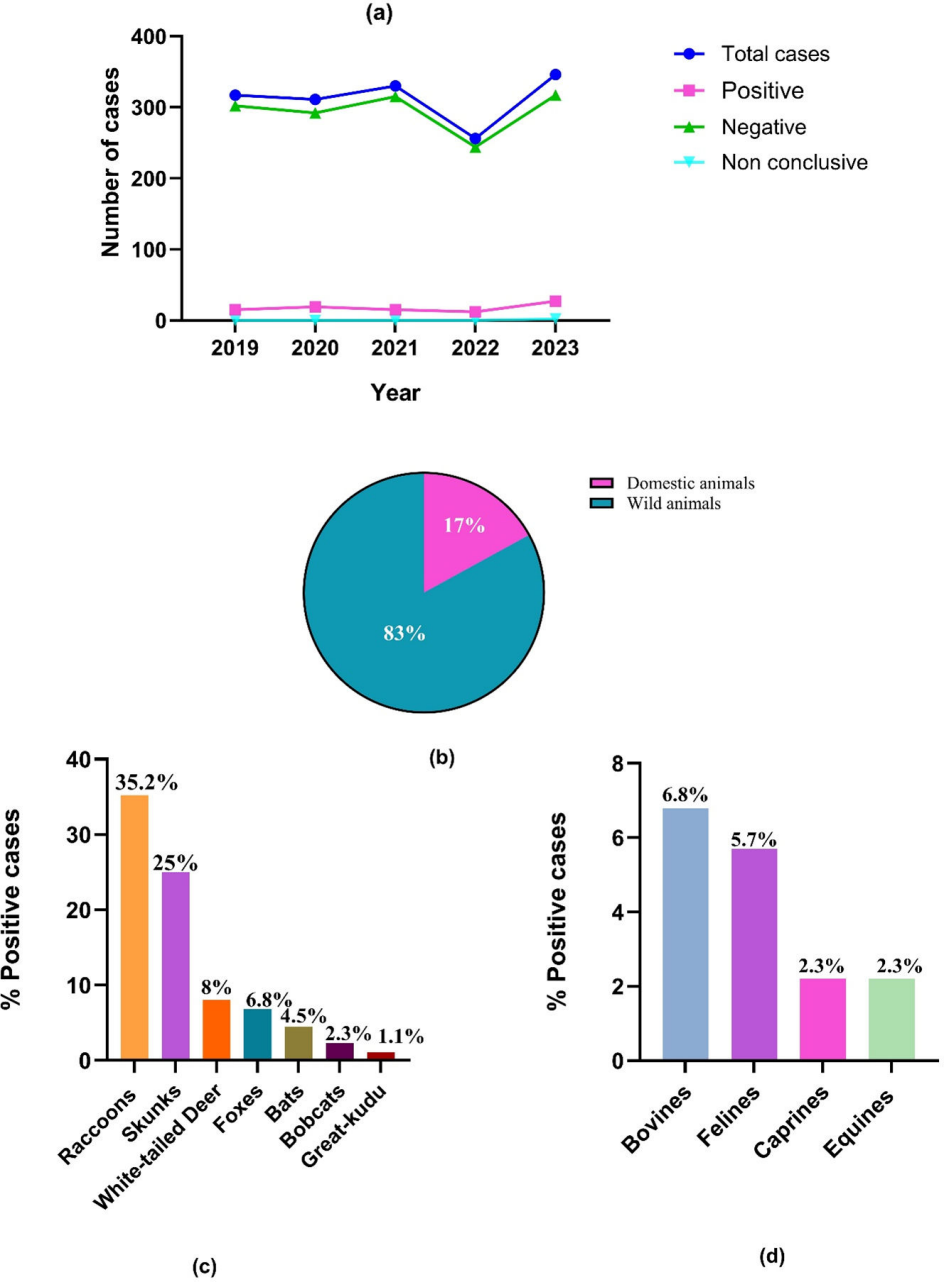
(**a**) Year-wise distribution of rabies from 2019 to 2023 from cases submitted at Athens Veterinary Diagnostic Laboratory (AVDL). (**b**) Distribution of positive rabies cases among domestic and wildlife animals over 5 years (2019–2023) from cases submitted at AVDL. (**c**) Wildlife species distribution of rabies over 5 years (2019–2023) from cases submitted at AVDL. (**d**) Domestic species distribution of rabies over 5 years (2019–2023) from cases submitted at AVDL.

A total of 1,560 cases were submitted for rabies testing from 2019 to 2023. Out of 1,560 cases, 94.2% [1,470/1,560] were negative for rabies, 5.6% [88/1,560] were positive for rabies across 11 species, and 0.1% [2/1,560] were non-conclusive cases. The occurrence of rabies varied between wildlife (83% [73/88]) and domestic (17% [15/88]) animals, and most rabies-positive cases were coming from Georgia. Over the 5 years, the negative and the total number of rabies submitted cases remained relatively stable until 2021, when there was a decrease followed by an increasing trend starting in 2022. The number of positives also remained stable, with a slight peak in 2022 (Fig. 1b). Wildlife species significantly accounted for most of the positive cases, with 83% (73/88) representing seven species, whereas 17% (15/88) were from domestic animals (*P*-value < 0.0001) (Fig. 1b). As shown in Fig. 1c, among wildlife, the affected species were raccoons (35.2% [31/88]), skunks (25% [22/88]), white-tailed deer (8% [7/88]), foxes (6.8% [6/88]), bats (4.5% [4/88]), bobcats (2.3% [2/88]), and great kudu (1.1% [1/88]). In domestic animals, the affected species were bovine (6.8% [6/88]), feline (5.7% [5/88]), caprine (2.3% [2/88]), and equine (2.3% [2/88]) (Fig. 1d).

The positivity rates also differ by species. Skunks (38.6% [22/57]) had the highest positivity rate among wildlife species, followed by great kudu (20% [1/5]), raccoons (12.3% [31/253]), foxes (10.7% [6/56]), bobcats (8.3% [2/24]), bats (7.8% [4/51]), and white-tailed deer (6.5% [7/107]). The domestic species, including bovine (13.3% [6/45]), equine (8.3% [2/24]), caprine (4.3% [2/47]), and feline (4.1% [5/121]), also displayed a difference in positivity rates.

**Fig 2:**
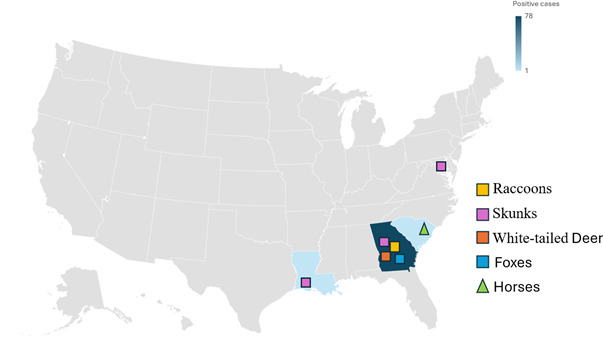
Geographical distribution of positive rabies cases received across the United States.

**Fig 3:**
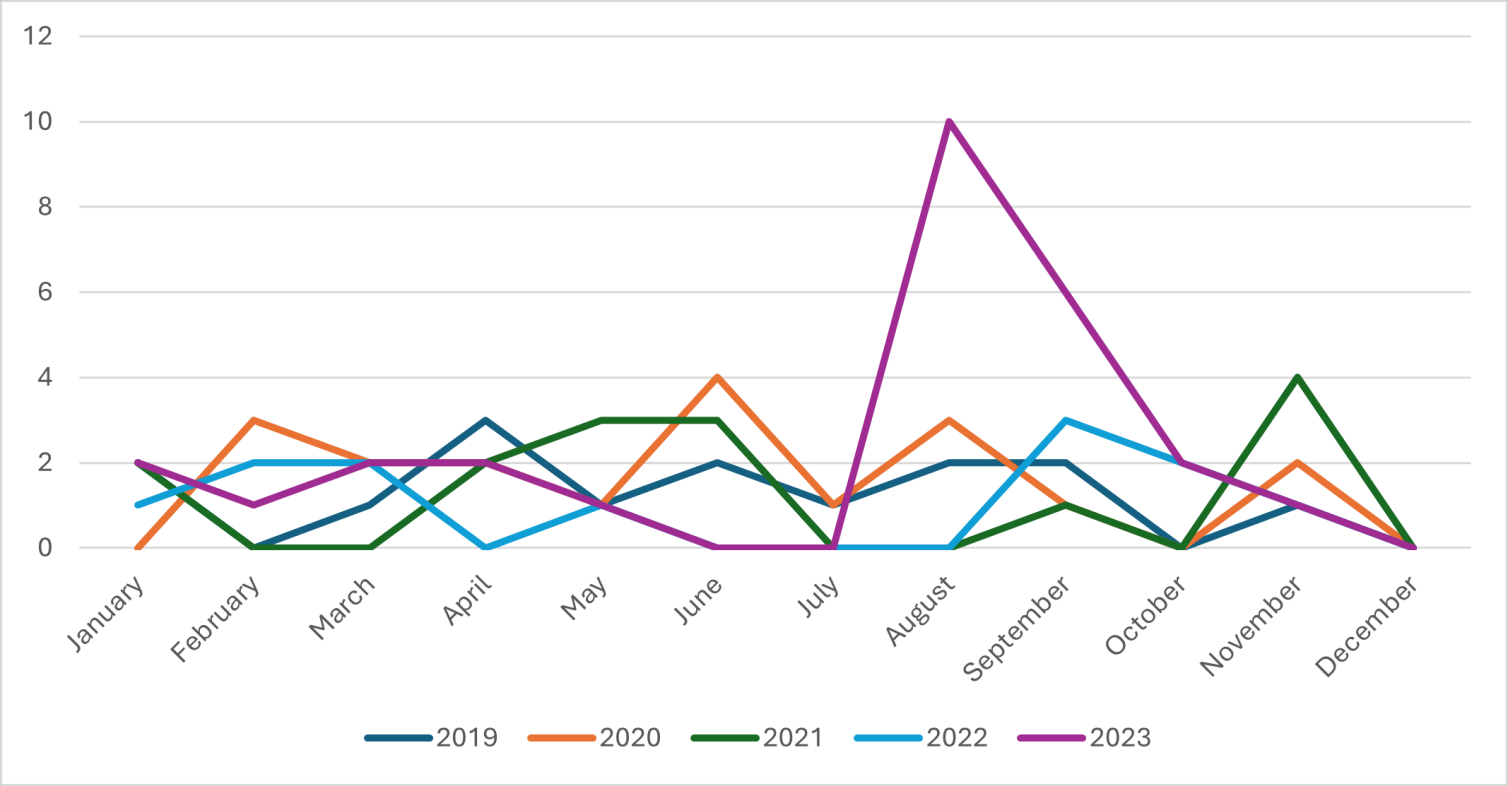
Seasonal trend of positive animal rabies cases per year (2019–2023) from cases submitted at AVDL. Seasons were defined as Winter (December–February), Spring (March–May), Summer (June–August), and Fall (September–November).

Figure 2 shows the distribution of rabies cases across most Southeastern states and Washington, D.C. Positive cases were predominantly located in Georgia (88.6% [78/88]), with additional cases identified in Washington, D.C. (2.3% [2/88]), Louisiana (1.1% [1/88]), and South Carolina (1.1% [1/88]). The remaining positive cases were reported with unknown locations (6.8% [6/88]). Additionally, we observed a seasonal variation in positive cases throughout the years, with noticeable peaks occurring during certain months. In 2023, specifically, the results showed a very sharp peak characteristic of an increase in positive cases from July until October (Fig. 3). The infection rates during fall (6.9% [26/379]), spring (6% [23/382]), and summer (5.7% [26/456]) were higher than in winter (3.8% [13/343]). The highest proportion of submitted cases was observed during the summer (29.2% [456/1,560]), and 24.3% [379/1,560], 24.5% [382/1,560], and 22% [343/1,560] accounted for submissions received during the fall, spring, and winter, respectively. There is, however, no significant statistical association between seasons and occurrence of rabies cases (*P*-value = 0.3387).

## DISCUSSION

The study investigated the occurrence of rabies among wild and domestic animals between 2019 and 2023, identified the most affected species, mapped the geographic distribution of positive cases, and analyzed rabies trends across the years, considering four different seasons (winter, spring, summer, and fall). In recent years, studies conducted in the United States have reported a significant decrease in the number of rabies-positive cases in 2021, followed by an increase in 2022 (28, 29). Our data reveal a comparable trend over the same period, with the decline observed in 2021 potentially related to decreased rabies surveillance activities during the COVID-19 pandemic (28). Our findings also showed that wildlife species exhibit a significantly higher occurrence of rabies than domestic animals, with 83% of cases (73 out of 88 total cases) reported in wildlife. Likewise, studies conducted in the southeastern United States also observed a higher occurrence of rabies in wildlife compared with domestic animals (30, 31). This higher occurrence in wildlife could be attributed to factors such as larger wildlife population densities, increased interactions between wildlife species, and habitat changes (32, 33). The roles of these factors in the occurrence of rabies were not assessed in this study, which is a limitation. Another factor could be a large sample size, especially for raccoons (*n* = 253), and the absence of wildlife-targeted vaccination programs in the southeastern United States. In our study, 79 out of 88 positive domestic and wildlife total cases had unknown vaccination history, which suggests a gap in the surveillance of rabies. Recently, challenges in administering oral rabies vaccination (ORV) in skunk populations have been reported (34). Therefore, further investigations could help in implementing more effective control measures, particularly for species like raccoons and skunks, which are known carriers of rabies. In contrast to our findings, domestic animals such as dogs and cats were reported as the most affected by rabies in Brazil, Ukraine, and South Africa (35–37).

The high occurrence of rabies in Georgia might be due to the proximity of our laboratory, where we may have received more cases within the state of Georgia than outside the state. This finding can also reflect the presence of RABV in this specific region and help inform targeted rabies control strategies. However, although the geographic distribution in our study was predominantly concentrated in Georgia, the additional cases identified in Florida and Alabama in another study suggest a broader regional spread of the disease across the southeastern United States (30).

Although no statistical significance was observed *(P = 0.3387)*, the rabies infection rates during the fall (6.9%), spring (6%), and summer (5.7%) seasons were higher than the winter (3.8%) seasons, aligning with the highest submission rates. It could be linked to a possible connection between increased activity among wildlife and domestic animals during warmer months and the higher occurrence of rabies (32). During the seasonal peak in the occurrence of rabies that we observed between July and October 2023, the most affected species were raccoons (7.6% [10/131]), whereas tailed deer (2.3% [3/131]), skunks (1.5% [2/131]), bats (0.8% [1/131]), caprine (0.8% [1/131]), and bovine (0.8% [1/131]) were the least affected. It is unknown whether this increase in rabies occurrence was due to a decrease in surveillance activity. In Brazil, equine rabies cases were consistently reported throughout the year, with no clear seasonality, although peaks were noted in certain months due to increased animal interactions (38). It is noteworthy that climate change, with its associated rise in temperature, has been associated with increased rabies cases since animals will be more active and be able to move longer distances in warmer temperatures, thereby potentially spreading the virus to other animals and even humans (32, 39). Moreover, our data confirm that wild animals are more likely to test positive for rabies than domestic animals in the southeastern United States. The most affected species, including raccoons, skunks, white-tailed deer, foxes, bats, and bobcats, further emphasize the significant role of wildlife in the circulation of RABV infection in the region.

Although DFAT is the gold standard for rabies testing (40), it has several limitations, including the high rate of inaccurate results due to the requirement of using high-quality brain samples to perform the test. Additionally, the interpretation of the results is very subjective and heavily depends on technicians who need to be highly trained before performing the test while following strict biosafety procedures (41). Access to a necropsy facility is required for proper collection of brain tissues, and a cold chain needs to be maintained to prevent degradation. Such challenges limit DFAT’s use, especially in resource-limited settings. Although the quality of submitted samples was not a constant constraint in our DFAT workflow, it is important to highlight these limitations when analyzing rabies data, especially collected in remote areas. Alternative methods, such as real-time polymerase chain reaction (RT-PCR) or LN34 Pan-Lyssavirus RT-PCR assays, could bridge the gap in point-of-care testing as they offer not only a very sensitive and specific diagnostic platform for RABV but also a rapid and cost-effective solution (42, 43).

Overall, our findings highlight distinct seasonal and geographical trends and the burden of rabies among various animal species. We expect these results would add valuable insights into the literature and public health policymakers in the Southeastern United States and contribute to the battle against rabies.
